# Using a Supramolecular Approach to Engineer Modular Hydrogel Platforms for Culturing Protoplasts – from General Tissue Engineering to Cellular Agriculture

**DOI:** 10.1002/adbi.202400690

**Published:** 2025-06-04

**Authors:** Maritza M. Rovers, Erik J. Slootweg, Ferdinand C. O. Los, Patricia Y. W. Dankers

**Affiliations:** ^1^ Institute for Complex Molecular Systems Eindhoven University of Technology P.O. Box 513 Eindhoven 5600 MB The Netherlands; ^2^ Department of Biomedical Engineering Laboratory of Chemical Biology Eindhoven University of Technology P.O. Box 513 Eindhoven 5600 MB The Netherlands; ^3^ Hudson River Biotechnology Nieuwe Kanaal 7V Wageningen 6709 PA The Netherlands; ^4^ Department of Chemical Engineering and Chemistry Eindhoven University of Technology P.O. Box 513 Eindhoven 5600 MB The Netherlands

**Keywords:** cell wall regeneration, cellular agriculture, protoplast regeneration, supramolecular hydrogels, tobacco BY‐2

## Abstract

Protoplast regeneration into plant cells and further into plants is an ongoing challenge in agricultural biotechnology. Inspired by mammalian tissue engineering, a strategic shift is proposed in plant tissue engineering to steer protoplast culture using fully synthetic materials‐based culture platforms. Here a supramolecular materials method to engineer modular culture methods for protoplasts is chosen to use. Supramolecular monomers as modular building blocks allow to make various hydrogel formulations and to study different protoplast cultures; including 2D cultures on top of supramolecular hydrogels, 2.5D cultures using supramolecular fibers in solution, and 3D cultures when encapsulated in bulk hydrogels or microgels. Importantly, the need is shown for bioactive functionalization of the supramolecular hydrogels with a peptide additive in 2D protoplast cultures. After 11 days, the bioactive hydrogel induced protoplast enlargement, which is absent on pristine hydrogels. The opposite effect is present for protoplasts cultured in 3D, showing plasmolysis as a result of the bioactive additive. Interestingly, in 2.5D lower bioactive additive concentrations in supramolecular fibers stimulated protoplast enlargement, demonstrated by similar morphological changes as in 2D. Finally, protoplast encapsulation in supramolecular microgels is showcased. This work demonstrates the potential to modularly engineer various synthetic platforms to facilitate cellular agriculture.

## Graphical abstract

Using supramolecular monomers, various hydrogel culture systems were formulated to culture protoplasts; including 2D, 2.5D, and 3D hydrogels. Depending on the culture platform, bioactive functionalization led to protoplast enlargement (2D and 2.5D) or plasmolysis (3D). This work shows the potential to modularly engineer synthetic platforms for cellular agriculture.

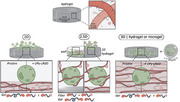

## 1 Introduction

The increasing global demand for agricultural crops due to world population growth and climate change necessitates innovative plant breeding methods like genome editing and targeted mutagenesis to enhance productivity and ensure food security.^[^ ^1^, ^2^, ^3^ ^]^ Genetic modifications are commonly introduced into protoplasts, which are single plant cells from which the supportive cell wall has been enzymatically or mechanically removed.^[^ ^4^, ^5^, ^6^ ^]^ Protoplast regeneration is a crucial step in gene‐editing plants, yet the bottleneck of the process.^[^ ^5^, ^6^, ^7^ ^]^

As of now, the golden standard to efficiently develop multicellular plant structures from modified single protoplasts has not been identified yet. This challenge is compounded by the fact that the process is highly specific to each plant type, hindering its uniform application across different species.^[^ ^5^ ^]^ Extensive research has been conducted on the impact of growth medium, temperature, and light on protoplast development.^[^ ^7^ ^]^ Additionally, different culture methods can exert significant effects on protoplast division and microcallus formation. Broadly, protoplasts can be cultivated either free in suspension or embedded in a matrix, where the latter one prevents protoplast agglutination.^[^ ^7^, ^8^, ^9^ ^]^ Usually, this matrix is a hydrogel, composed of polysaccharides such as alginate or agarose.^[^ ^10^ ^]^

The utilization of alginate and agarose embedding techniques for protoplast regeneration has been extensively employed, with the choice often depending on the specific plant species involved.^[^ ^10^ ^]^ In addition, protoplast regeneration is not only influenced by the type of matrix, but was also affected by in what form this matrix was provided to the cells. For example, conventional 3D hydrogel encapsulation may hinder downstream cellular processes and may cause negative cellular waste accumulation during culture.^[^ ^11^ ^]^ For this reason, numerous studies on protoplast regeneration have explored various culture methods based on alginate or agarose, including liquid suspension culture, suspension over a rigid substrate, suspension over a hydrogel layer, or protoplast embedded in the hydrogel. For example, Sinha and co‐workers showed that for lupin protoplasts embedded in alginate failed to regenerate, while those embedded in agarose droplets did trigger mitosis.^[^ ^8^ ^]^ The protoplasts were suspended over an agarose layer, embedded in a single agarose layered hydrogel, or encapsulated within agarose droplets, which was the most promising condition. Differently, Pati et al. developed an extra thin alginate film (ETAF) as an improved version of the thin alginate layer (TAL) developed by Golds and co‐workers.^[^ ^12^, ^13^ ^]^ The TAL technique already advanced earlier methods, including improved cell tracking, ease of transfer to fresh medium, decreased release of toxic substances, and a notable reduction in time and steps from protoplast isolation to regeneration. The ETAF further improved this by allowing easier cell observation, cost‐reduction, and increased plating efficiency and protoplast regeneration.

For decades, hydrogels derived from agarose and alginate have been investigated and applied as matrices for protoplast cultures. Despite their inherent versatility in facilitating diverse culture methods, the hydrogel composition, including factors like stiffness, porosity, and degradability, is intrinsic to the utilized macromolecular building blocks.^[^ ^14^ ^]^ Furthermore, using these natural resources can bring issues such as batch variability.^[^ ^10^ ^]^ This challenge is further compounded by substantial variations in cellular requirements among different plant species and the fact that the relationship between these hydrogel matrix properties and the cellular protoplast behaviour is not completely understood yet.^[^ ^7^, ^10^, ^11^, ^15^ ^]^ The need for novel hydrogel systems based on, for example, carefully designed synthetic polymers is suggested.^[^ ^10^ ^]^

**Figure 1:**
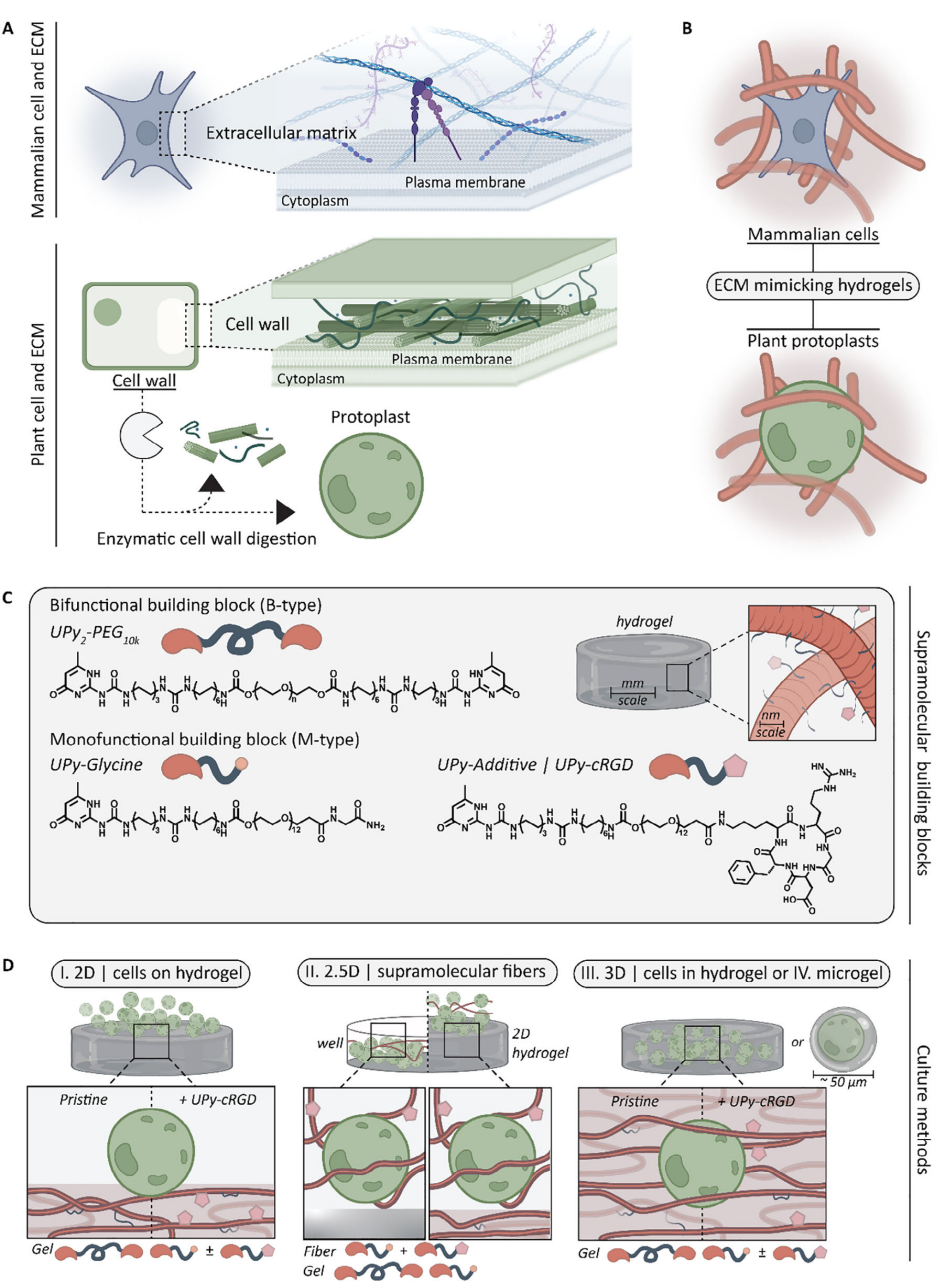
Aim and molecular design of this study. A) The extracellular matrix (ECM) in animal cells and the cell wall in plant cells share a common architectural feature. In plants, it is possible to extract the cell wall through enzymatic digestion, resulting in the isolation of protoplasts. B) The shared similarities between mammalian and plant ECMs suggest that synthetic hydrogel design in mammalian tissue engineering principles might be relevant for protoplast culture. C) Supramolecular monomeric building blocks used in this study: B‐type (UPy_2_‐PEG_10K_; n ≈227) and M‐type (UPy‐glycine), including UPy‐cRGD additive. D) These building blocks can be formulated into hydrogels, allowing various culture methods, including I. 2D protoplast culture on gels, II. 2.5D in coculture with supramolecular fibers, or III. 3D encapsulated in gels or IV. microgels.

To our knowledge, synthetic hydrogels for protoplasts remain unreported, mammalian tissue engineering and regenerative medicine have already established this approach, employing many synthetic hydrogel systems, e.g., based on poly(ethylene glycol) (PEG), polyacrylamide (PAM), and polyvinyl alcohol (PVA). These hydrogels were used to mimic the physical environment of cells, known as the extracellular matrix (ECM), to promote regeneration and growth and to create multicellular structures (e.g., organoids) from single cells.^[^ ^16^, ^17^, ^18^ ^]^ Therefore, synthetic hydrogels are designed to match 1) mechanical and 2) biological properties of the ECM.^[^ ^19^ ^]^ Similarly, in plants, the extensive cell wall fulfils the role of ECM and is involved in numerous processes; it provides flexibility to support cell division, acts as a biochemical scaffold that enables differentiation, and forms a protective barrier against biotic and abiotic stress.^[^ ^20^ ^]^ The shared similarities between mammalian and plant ECMs suggest that design principles for synthetic hydrogels might be relevant for protoplast culture too (**Figure** **1**A,B).

First, environmental mechanical forces or deformations are sensed by mammalian cells via the ECM and converted into biological responses. In plant cells, mechanical forces and stress are balanced between cell wall tension and turgor pressure. The mechanics of the cell wall shifts dynamically over time and space, adapting to developmental and stress signals.^[^ ^21^ ^]^ It is responsible for the intrinsic origin of stress in plants, influencing migration, growth, development, and morphogenesis.^[^ ^22^ ^]^ Hydrogels could be designed to mimic the mechanical forces experienced by the plant cell through the cell membrane. Second, for mammalian cells, hydrogels are often modified by incorporating ECM‐mimicking peptides or proteins to replicate the biological signalling properties of the ECM.^[^ ^23^ ^]^ Similarly, it is hypothesized that plant cells may also possess common biochemical moieties that link the plant cell membrane to the cell wall.^[^ ^24^ ^]^ One such example is the arginine‐glycine‐aspartate (RGD) peptide, which plays a fundamental role in cell adhesion in both animals and plants.^[^ ^25^ ^]^ In mammalian ECM, the RGD is an integrin‐binding domain present in ECM proteins that cells can bind via plasma membrane receptors, which is crucial for cell attachment to the ECM, as well as spreading and migration.^[^ ^26^ ^]^ While plants do not have integrin proteins in their cell wall like animals, Langhans et al. have identified specific subdomains in plants that exhibit integrin‐like proteins.^[^ ^27^ ^]^ These proteins share homologies with certain regions of human integrin domains and play a crucial role in mediating adhesion between the plasma membrane and the cell wall through protein‐receptor binding motifs.^[^ ^27^, ^28^ ^]^ This theory was confirmed in the study of Schindler et al., where exogenous RGD was added to various plant cell suspension cultures.^[^ ^29^ ^]^ They hypothesized that the exogenous RGD would compete with the endogenous RGD‐containing proteins in the cell wall. As a result, after the addition of exogenous RGD, growth alterations and inhibition in the formation of plasma membrane–cell wall connections were observed for suspension cultures of soybean and Arabidopsis, respectively. A similar phenomenon was observed in human cell studies, where the addition of soluble RGD to chondrocyte culture resulted in the inhibition of adhesion to fibronectin in the ECM.^[^ ^30^ ^]^ In contrast, the presence of RGD‐peptides during the growth of maize calluses resulted in heightened embryogenesis compared to calluses grown in the absence or with the control peptide RGE.^[^ ^31^ ^]^ Canut and colleagues examined the effect of RGD on onion epidermal cells and Arabidopsis cultured cells. Significant alterations occurred in both cell types after treatment: in onion cells, the Hechtian strands connecting the cell wall to the membrane were lost, while Arabidopsis cells plasmolysed.^[^ ^32^ ^]^ Additionally, they proved the attachment of Arabidopsis protoplasts to a genetically engineered protein with RGD‐motifs.

Taken together, these observations provide strong evidence for the existence of integrin‐like proteins in plants, suggesting the potential benefit of including RGD‐moieties in the synthetic hydrogel.

While synthetic hydrogels offer greater tunability to meet the above criteria, it is recognized in mammalian tissue engineering that they often exhibit disparities in both structure and composition compared to native tissue.^[^ ^33^ ^]^ This is caused because usually these synthetic hydrogels are based on covalent interactions, while the mammalian ECM and the cell wall both consist of non‐covalent interactions.^[^ ^34^, ^35^, ^36^ ^]^ In plants, lignin and hemicellulose assemble into a complex supramolecular network around the cellulose fibrils, where the non‐covalent interactions play a crucial role in locally modulating the cell wall properties and retaining the stress in the wall, while allowing deformations when cells grow.^[^ ^21^ ^]^ For this reason, synthetic hydrogels based on non‐covalent interactions might be a more suitable candidate for protoplast regeneration, as it is most suited to closely mimic the structure of the natural cell wall.

Synthetic hydrogels, based on non‐covalent interactions, can be formulated using supramolecular building blocks, which are defined as assemblies formed from a single type or multiple types of molecules via directional and reversible non‐covalent bonds.^[^ ^37^, ^38^ ^]^ In general, synthetic supramolecular systems have already been well explored as culture substrates for mammalian cells, such as self‐assembling ultrashort peptides (SUPs) and peptide amphiphiles (PA).^[^ ^39^, ^40^ ^]^ An additional advantage of using supramolecular building blocks is that we can easily tune the material properties, especially when the supramolecular units are modular, like for the bis‐urea (BU), benzene‐1,3,5‐tricarboxamide (BTA), and ureido‐pyrimidinone (UPy) moieties.^[^ ^41^, ^42^, ^43^ ^]^ Considering the beneficial role of tunability in hydrogel for transitioning these hydrogel systems from mammalian to plant cell culture, using modular supramolecular hydrogels is of great interest.

In our lab, we have great expertise with UPy‐moieties as a supramolecular building block. These units dimerize via quadruple hydrogen bonding and stack laterally using *π*–*π* interactions. Introducing an alkyl spacer shields water, promoting assembly into hierarchical fibers.^[^ ^44^, ^45^, ^46^ ^]^ Diba and coworkers have demonstrated that hydrogels can be formulated through a two‐component system comprising mono‐ and bifunctionalized UPy‐monomers (referred to as M‐ and B‐type molecules, respectively) (Figure 1C).^[^ ^47^ ^]^ The modularity of the system provides a range of tunable material properties, e.g., stiffness, dynamics, bioactivity, and the ability to alter them independently.^[^ ^47^, ^48^, ^49^ ^]^ This has proven to facilitate the cultivation of diverse cell types, serving as models for various mammalian organs, including cornea‐derived keratocytes, Madin–Darby canine kidney (MDCK) cells, human vena saphena cells (HVSC), human renal proximal tubule epithelial cells (RPTECs), human normal dermal fibroblasts (hNDFs), and cardiomyocyte progenitor cells (CMPCs).^[^ ^47^, ^48^, ^49^, ^50^, ^51^ ^]^ Here, we propose to further expand our hydrogel system to support the culture of plant protoplasts.

The aim of this research is to translate our supramolecular UPy‐based hydrogel system, originally designed for mammalian tissue engineering, to the field of protoplast culture and regeneration for cellular agriculture. In that regard, fresh protoplasts enzymatically derived from *Nicotiana tabacum* L. cv. Bright Yellow 2 (tobacco BY‐2) cells were used as a model system.^[^ ^52^ ^]^ Hydrogels incorporating M‐ and B‐type UPy‐moieties were formulated, and their performance on protoplast cultures was evaluated by analyzing protoplast size expansion and circularity. The influence of RGD on the protoplasts during culture was investigated by integrating a functionalized UPy with a cyclic variant (UPy‐cRGD) into the hydrogel. The hydrogels can be used as various culture methods: formulated as substrates where protoplasts are cultured in 2D on top of the gel, or protoplasts can be encapsulated within the 3D hydrogel matrix, both conditions with and without UPy‐cRGD (Figure 1D). Also, in 2.5D hydrogels, a diluted state can be added to the cells on top of a hydrogel as supramolecular fibers. The regenerative capacity of the hydrogel and culture method was evaluated based on the protoplasts' morphological changes, expansion in size, and circularity. Finally, to better emulate the native microenvironment formed by the cell wall, we can downsize our hydrogel system to a microscopic scale, forming microgels comprising only a few picolitres.^[^ ^53^ ^]^ These microgels can be produced using droplet‐based microfluidics, enabling the encapsulation of single protoplasts within microgels. In this setup, the protoplast will be surrounded by a thin layer of hydrogel, akin to how the cell wall envelops the cell membrane.

## 2 Results and Discussion

### 2.1 Protoplast Isolation and Material Formulation

**Figure 2:**
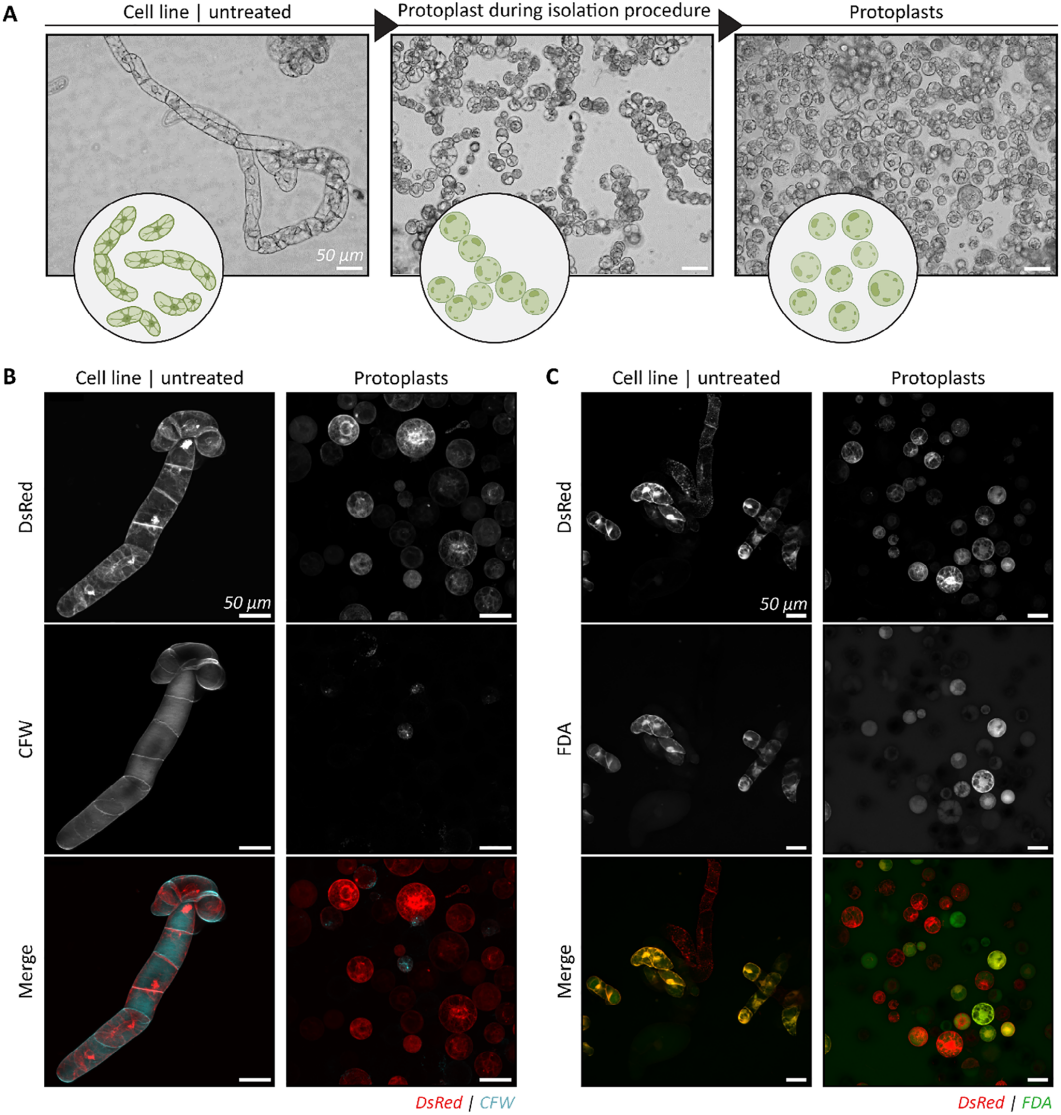
DsRed modified tobacco BY‐2 cells were used as a model cell line. A) Untreated cells exhibit chain‐like structures. Single protoplasts were obtained by enzymatically digesting the cell wall. Protoplasts appear round and connected before isolation, while after cell work‐up, single protoplasts were obtained. B) The cell wall was present in untreated suspension cells, stained by Calcofluor White (CFW), and digested in the protoplasts. CFW in cyan and DsRed in red. C) Cell viability was assessed by the fluorescein diacetate (FDA) staining. FDA in green and DsRed in Red. All scale bars represent 50 µm.

Tobacco BY‐2 protoplasts isolation was based on an adapted previous reported study by Lei et al., and Negrutiu and coworkers.^[^ ^54^, ^55^ ^]^ Enzymatic digestion based on 1.0 w/v% cellulase R‐10, 0.5 w/v% macerozyme R‐10, and 0.1 w/v% pectolyase was used to remove the cell wall. Confocal microscopy images confirmed the successful removal of the cell wall in protoplasts, evident by the absence of Calcofluor White (CFW) staining that typically binds to cellulose and chitin present in the cell wall (**Figure** **2**A,B). The protoplast isolation procedure was cell‐friendly as exhibited by a positive viability fluorescein diacetate (FDA) staining (Figure 2C; Figure S1, Supporting Information).

Supramolecular hydrogels based on M‐ and B‐type molecules were formulated by dissolving the individual building blocks at predetermined concentrations in an aqueous solution and subsequently diluted with regeneration medium to attain the final concentration of both components at pH 5.8. For 2D culture, the hydrogelators were mixed, and after proper gelation, protoplasts were added on top of the gel. For 3D culture, protoplasts were carefully mixed into the B‐type solution. Upon addition of the M‐type solution, a protoplast‐encapsulated hydrogel forms. Lastly, hydrogels in a diluted state can be added to the protoplast to investigate the influence of supramolecular fibers on the protoplast growth. For all experiments, protoplasts in a well with regeneration medium served as a control group.

### 2.2 Supramolecular (2D and 3D) Hydrogels to Support Protoplast Culture

**Figure 3:**
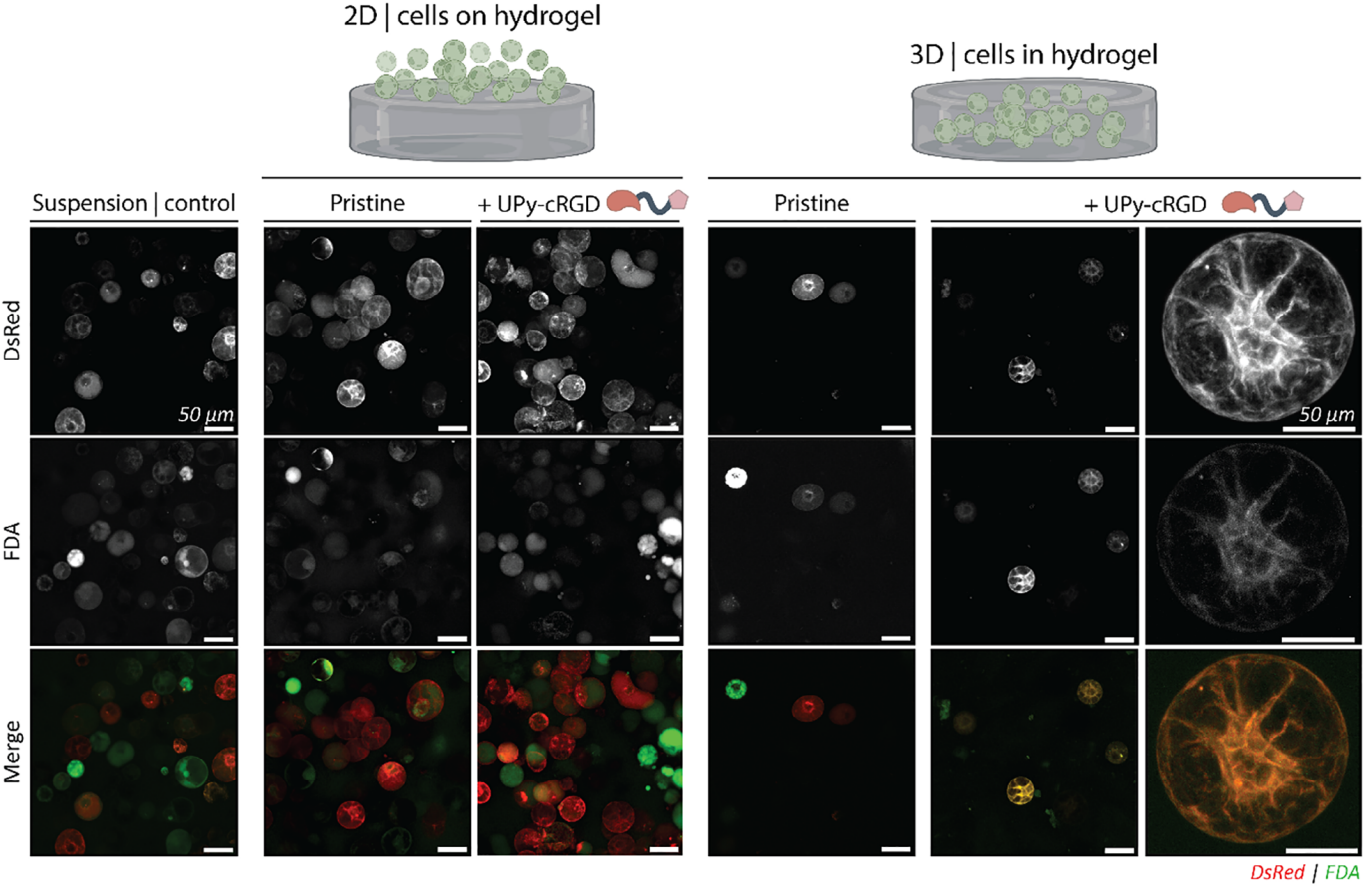
FDA staining of BY‐2 protoplasts cultured on 2D and in 3D hydrogels. Protoplasts cultured in suspension, 2D cultured on hydrogels, and embedded 3D in a hydrogel. Viability was evaluated 24 h post‐cultivation by a fluorescein diacetate (FDA) staining. For all conditions, the FDA in green overlapped with the DsRed protein in red present in the protoplasts, which suggests no influence of the culture methods on the protoplast viability. All scale bars represent 50 µm.

Protoplasts were cultured in 2D on top or 3D inside the supramolecular hydrogels, and the protoplasts viability and morphological changes were investigated over time (Figure S2A, Supporting Information). These hydrogels were either unfunctionalized (pristine) or made bioactive with a UPy‐cRGD additive. Cell viability was examined using an FDA staining on all conditions after a culture period of 24 h. Both cells cultured in 2D and 3D exhibited positive staining for FDA, suggesting that the embedding procedure itself is non‐detrimental to protoplasts (**Figure** **3**A). However, only the protoplasts within the pristine 3D hydrogel exhibited a lower FDA percentage of the total protoplast population compared to the control group (37.5% vs 49.4%, respectively; Figure S1, Supporting Information).

**Figure 4:**
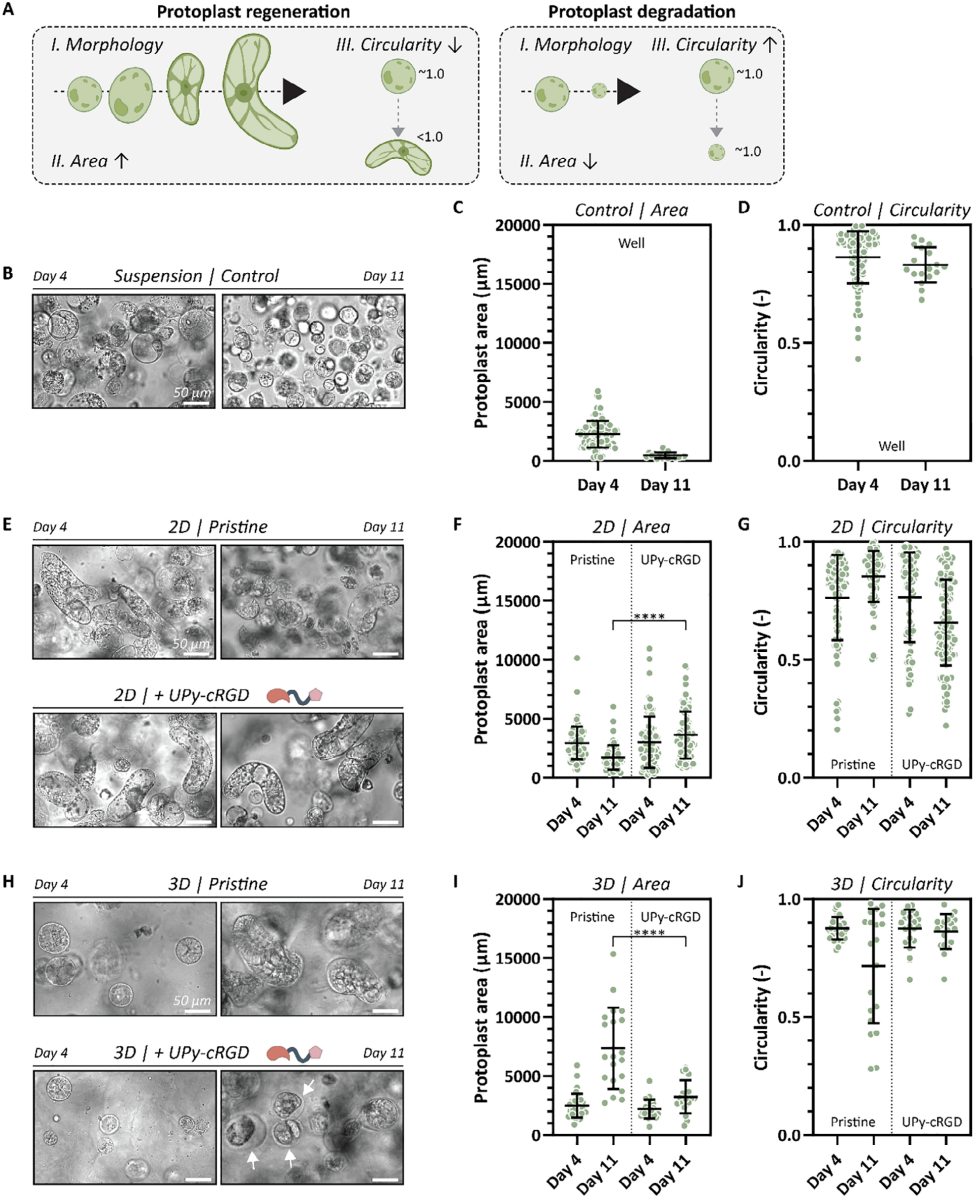
The regeneration potential of protoplasts cultured in 2D and 3D was assessed by morphological changes from spherical to a chain‐like shape at day 4 and 11. A) Here, protoplast regeneration is defined as the morphological transition of protoplasts from a spherical shape to an elongated form, with an increased area and decreased circularity, while protoplast degradation leads to shrinking and retaining a round shape. B) Cells cultivated in liquid medium kept their spherical morphology. A reduction in C) size was observed along with a high D) circularity, signifying a deficiency in protoplast regeneration after 11 days. E) In 2D culture, protoplasts increased in size and elongated with the addition of UPy‐cRGD, showing a significant increase in F) size and a decrease in G) circularity compared to pristine hydrogels after 11 days. H) In pristine and UPy‐cRGD functionalized 3D culture, protoplasts increased in size. A significant increase in I) size and a decrease in J) circularity were observed for pristine hydrogels compared to functionalized hydrogels after 11 days. All scale bars represent 50 µm.

Then, the protoplasts were investigated for their regenerative potential by changes in morphology, protoplast expansion, and circularity using bright field microscopy at days 4 and 11. Here, we define protoplast regeneration as the morphological transition of protoplasts from a spherical shape to an elongated form, characterized by an increase in protoplast area (expansion) and a decrease in circularity (**Figure** **4**A). Conversely, protoplast degradation or death results in the opposite effect, with protoplasts shrinking in size while maintaining their round shape. Cells cultivated in liquid medium retained their spherical morphology at day 4 (Figure 4B). However, a discernible reduction in size was observed along with a high circularity, signifying a deficiency in protoplast regeneration after 11 days (Figure 4C,D). For protoplasts cultured in 2D, an augmentation in size along with a notable commencement of stretching and elongation (decrease in circularity) was observed, compared to the control cells after 11 days of culture (Figure 4E–G). No discernible distinctions were observed between protoplasts cultured on pristine hydrogels or with UPy‐cRGD functionalization on the fourth day. Nonetheless, cells started to form more elongated and bigger structures when cultivated on functionalized hydrogels, visible at day 11 (Figure 4F). This suggests a stimulative effect of the RGD on the protoplast growth. Protoplasts embedded in pristine 3D hydrogels exhibited similar spherical morphologies to those observed in the liquid control group at day 4 (Figure 4H). Elongation of these cells was observed at day 11 (Figure 4J). In contrast, protoplasts within UPy‐cRGD functionalized 3D hydrogels did not elongate or augment in size (Figure 4I,J). Additionally, as an effect of plasmolysis, detachment of the cellular membrane from the thin newly formed cell wall was observed at day 11 (white arrow; Figure 4H).

To assess cell number in the protoplast culture, we determined the normalized cell number of protoplasts cultured for 4 and 11 days under different conditions. Brightfield images were acquired at various magnifications (10× with 1.6× zoom, 20×, 20× with 1.6× zoom, and 40×) and normalized to the imaged area of the confocal 10× magnification. The resulting data were plotted as the normalized cell number per condition (Figure S3A, Supporting Information). The generally lower cell count in 3D hydrogels is likely due to the even distribution of protoplasts throughout the hydrogel, leading to fewer cells being captured within a single image frame. Over time, a decline in cell number was observed across all conditions. However, Figure 4F–I shows an increase in cell area for the 2D UPy‐cRGD and 3D pristine hydrogels, respectively. This reduction in normalized cell number may be attributed to the increased size and elongated morphology of the cells, making fewer of them visible within a single imaged focal plane.

Notably, a substantial difference was observed in 2D or 3D protoplast culture on hydrogels with an equivalent concentration of 500 µm UPy‐cRGD. Protoplasts cultured in 2D showed an increase in size and morphological transformation into chain‐like structures, whereas in 3D, they did not. First, the culture method itself could have a huge influence on the protoplast regeneration. Cells cultured in 2D are fully submerged in medium, which is favorable for oxygen and nutrient exchange. Although the cells in the control group cultured in medium only performed less, suggesting that the presence of the hydrogel does have a positive effect on the cells. Protoplasts embedded in a 3D hydrogel might be negatively influenced by inadequate aeration and nutrition exchange. These challenges are evident in both pristine and functionalized 3D hydrogels, as cells at the bottom of the well exhibited smaller and appeared less healthy (Figure S2B, Supporting Information). Second, the method of cultivation exposes the UPy‐cRGD different to cells. Despite both hydrogels for 2D and 3D culture containing 500 µm UPy‐cRGD, this additive might be sensed differently by the cells. We hypothesize that when cells are embedded in 3D hydrogels, they experience locally high concentrations of UPy‐cRGD, which causes plasmolysis. This phenomenon was also described in literature for liquid cultures in the presence of 0.25 mg mL^−1^ GRGDSP peptide (equates 420 µm), where exogenous GRGDSP would compete with the endogenous RGD‐containing proteins in the cell wall.^[^ ^56^ ^]^ While Zaban and colleagues reported that subjecting protoplasts to a lower concentration of 1 ng mL^−1^ heptapeptide YGRGDSP (equates 1.3 × 10^−3^ µm) enhances axis formation and manifestation in regenerating protoplasts.^[^ ^57^ ^]^ This might explain why cells on 2D showed similar axis manifestation as reported by the research of Zaban. It is hypothesized that the dynamic nature of the supramolecular hydrogel facilitates a gradual release of molecules over time, allowing them to interact with the cells. This leads to a prolonged, low‐concentration exposure to the UPy‐cRGD additive, thereby contributing to its favorable impact on the regenerative capacity of the cells. These results stress the importance of different culture methods, as similar hydrogel compositions can have an adverse effect on the protoplast regeneration when used for 2D or 3D culture.

### 2.3 Positive Effect of Supramolecular RGD‐Enriched Fibers on Protoplast Culture

**Figure 5:**
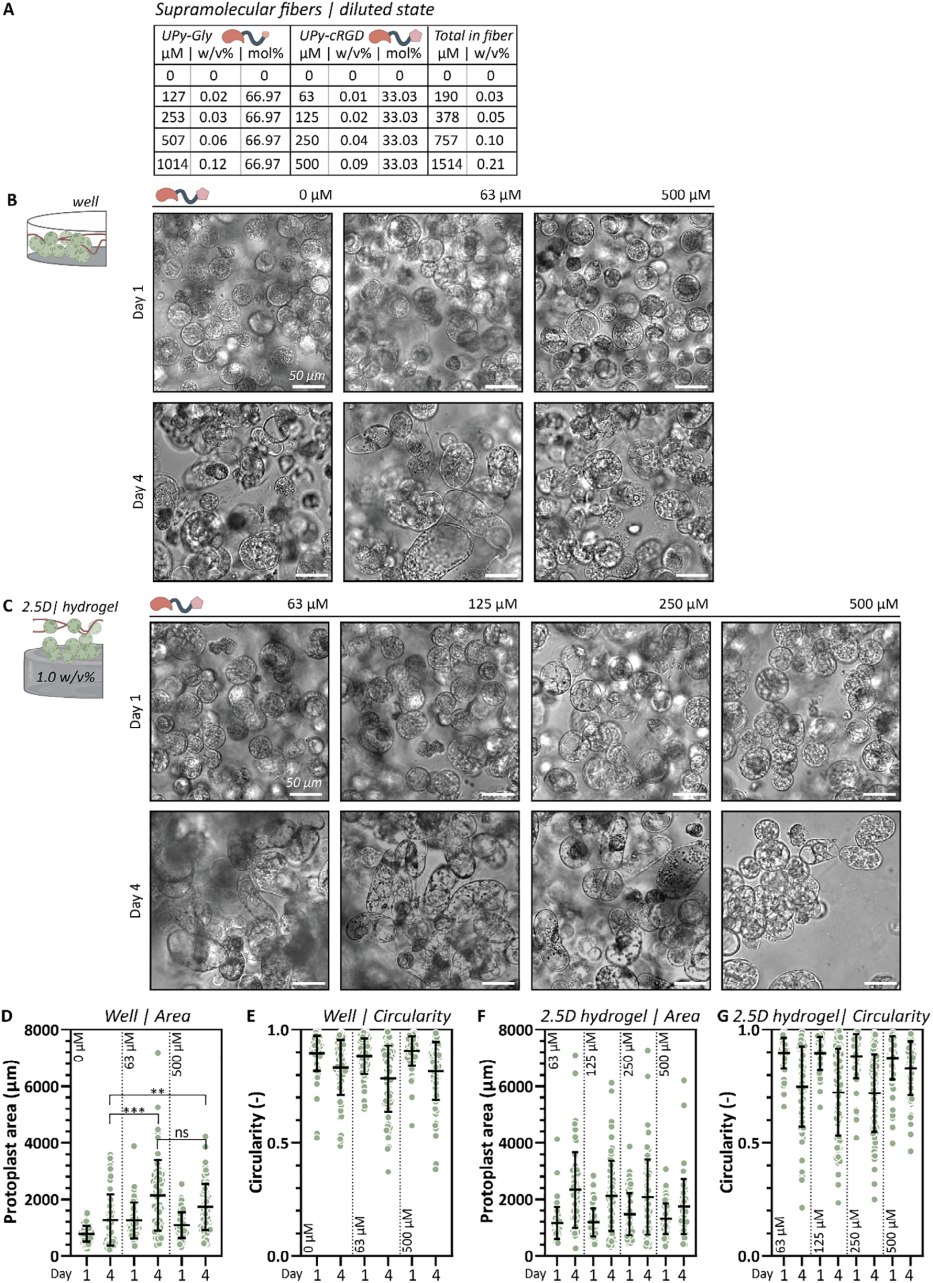
2.5D protoplast culture with UPy‐cRGD enriched supramolecular fibers in solution. A) Weight percentage and UPy‐cRGD content of the different formulated supramolecular fibers. B) Brightfield images of protoplasts cultured in a well with supramolecular fibers containing 0, 63, or 500 µM UPy‐cRGD imaged at day 1 and 4. C) Brightfield images of protoplasts cultured 2.5D: on a 1.0 w/v% hydrogel with supramolecular fibers containing 63, 125, 250, or 500 µm UPy‐cRGD imaged at day 1 and 4. Quantification of protoplast D) size and E) circularity cultured with 63 and 500 µm UPy‐cRGD enriched supramolecular fibers in a well. Quantification of protoplast F) size and G) circularity cultured with 63, 125, 250, or 500 µm UPy‐cRGD enriched supramolecular fibers on a hydrogel. All scale bars represent 50 µm.

The previously described results show the influence of different culture methods, either 2D or 3D, on the protoplast morphology and growth. To verify whether 2D culture promotes protoplast regeneration by gradually releasing UPy‐cRGD molecules over time, we cultured protoplasts in 2.5D in a well or on a pristine hydrogel with additional UPy‐cRGD enriched supramolecular fibers in solution. The fibers were formulated by dissolving M‐type UPy‐monomers along with UPy‐cRGD. Various concentrations were prepared by further diluting the fibers with regeneration medium (**Figure** **5**A; Table S2, Supporting Information). Initially, the protoplasts were cocultured with the fibers in a well plate and regeneration was evaluated based on protoplast growth (changes in protoplast area) and morphological changes from spherical to a chain‐like shape (decrease in circularity). No significant differences were discernible from the brightfield images taken between protoplasts cultured with 63 or 500 µm UPy‐cRGD enriched supramolecular fibers, and those without any fibers at day 1 (Figure 5B; Figure S4A, Supporting Information). However, protoplasts cultured with supramolecular fibers did show enlarged protoplasts compared to the control at day 4. This might indicate that a small addition of this UPy‐cRGD stimulated protoplasts to grow and elongate.

Next, protoplasts were cultured in 2.5D: on a 1.0 w/v% UPy‐based hydrogel along with supramolecular fibers containing 63, 125, 250, or 500 µm UPy‐cRGD (Figure 5C; Figure S4B, Supporting Information). Again, regeneration was assessed based on size and morphological changes after a culture of 1 and 4 days. No significant differences were observed in the brightfield images after 1 day. However, protoplasts cocultured with 63, 125, and 250 µm began to elongate and exhibit chain‐like structures, indicative of regeneration, at day 4. However, protoplasts cultured with 500 µm UPy‐cRGD in fibers did not display the elongated shape. Again, this indicates that a small addition of this UPy‐cRGD stimulated protoplasts to elongate.

These results show that a small addition of RGD to the culture via UPy‐cRGD functionalized supramolecular fibers can stimulate protoplast elongation. This was confirmed through quantitative analysis of protoplast area and circularity for cells cultured both in wells and on hydrogels with UPy‐cRGD‐enriched fibers. Fibers added to a well positively influenced protoplast size after 4 days (Figure 5D). However, no significant differences were observed between 63 and 500 µm UPy‐cRGD concentrations. Additionally, protoplast elongation increased in all cases by day 4 compared to day 1, as indicated by the decrease in protoplast circularity (Figure 5E). For fibers added to protoplasts on a hydrogel, protoplast size increased in all cases (Figure 5F). However, no significant differences were observed between UPy‐cRGD concentrations, although the 500 µm concentration appeared to less effectively stimulate protoplast elongation, as indicated by a higher circularity (Figure 5G).

To assess cell number in the protoplast culture, we determined the normalized cell number of protoplasts cultured for 1 and 4 days under different conditions. Brightfield images were acquired at various magnifications (10× with 1.6× zoom, 20×, 20× with 1.6× zoom, and 40×) and normalized to the imaged area of the confocal 10× magnification. The resulting data were plotted as the normalized cell number per condition (Figure S3B,C, Supporting Information). Over time, a decline in cell number was observed across all conditions. However, Figure 5D–G shows an increase in cell area for all conditions. This reduction in normalized cell number may be attributed to the increased size and elongated morphology of the cells, making fewer of them visible within a single imaged focal plane.

### 2.4 Protoplast Encapsulation in Supramolecular Microgels

**Figure 6:**
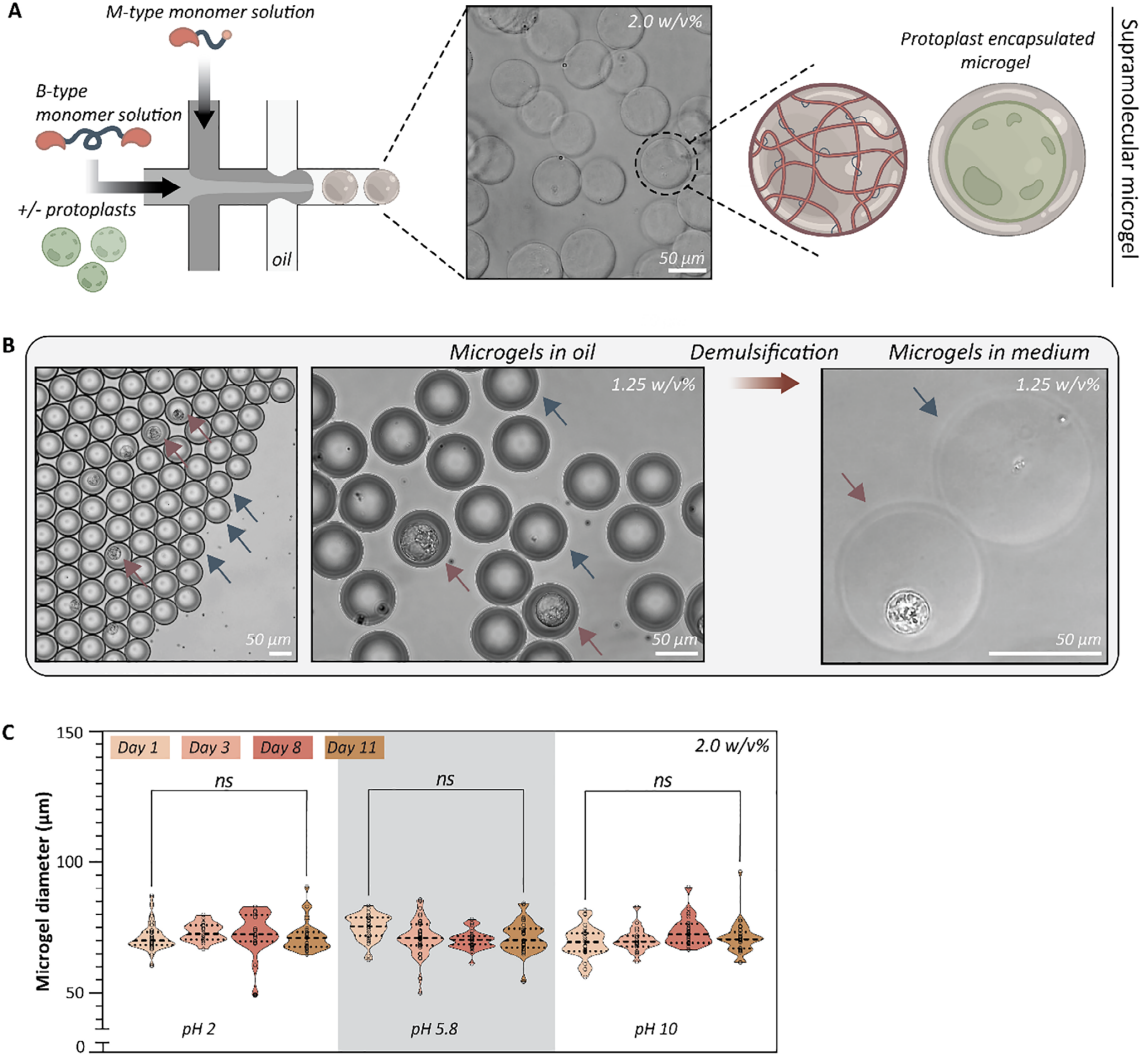
3D protoplast encapsulation in supramolecular microgels. A) Schematic representation of the microfluidic chip layout to formulate (cell‐encapsulated) microgels. Protoplasts can be encapsulated in the microgel by mixing them with the B‐type molecules. Brightfield image of 2.0 w/v% microgels isolated from the oil. B) Protoplasts encapsulated in 1.25 w/v% microgels in the oil and after demulsification in regeneration medium. Brown arrows represent encapsulated protoplasts, and blue arrows represent empty microgels. C) Microgels (2.0 w/v%) are considered stable by retaining their size at basic, acidic, and physiological conditions for a period of 11 days. All scale bars represent 50 µm.

Finally, to better emulate the native microenvironment formed by the cell wall, we downsized our hydrogel system to a microscopic scale, forming protoplasts encapsulated in microgels using droplet microfluidics. In the future, this method of protoplast culture could potentially facilitate the assessment of gene‐edited protoplasts at a single‐cell level without the requirement for manual separation. Therefore, as a proof of concept, M‐type and B‐type monomers were dissolved separately, and protoplasts were mixed in the B‐type hydrogelator for subsequent encapsulation in the microgel (**Figure** **6**A; Table S3, Supporting Information). Both M‐ and B‐type, whether with or without protoplasts, were separately introduced into a pipette‐tip‐based droplet microfluidic setup, where at the point of intersection, the hydrogelators combined in a 1:1 volume ratio, where the faster oil phase generated microdroplets from the aqueous polymer stream. The introduction of protoplasts in the B‐type hydrogelator ensures their encapsulation within the crosslinked polymer network. After proper gelation, microgels formulated at 1.25 w/v% exhibited sufficient stability, facilitating their isolation from the oil phase through demulsification (Figure 6B). Lastly, the stability of the microgels was tested by storing 2.0 w/v% microgels without protoplasts under acidic, physiological, and basic MilliQ water at 25 °C (Figure 6C). The stability of the microgels was evaluated in terms of diameter change over time. The statistical analysis revealed no significant deviation in diameter for microgels preserved in all three conditions for a period of 11 days, compared to their initial size.

## 3 Conclusion

Our research showed a synthetic, supramolecular approach to create modular culture platforms for protoplasts, highlighting the possibilities for cellular agriculture. In more detail, the addition of bioactive peptide additives showed to have different effects in the different culture methods, being 2D, 2.5D, and 3D, showing the importance of different formulation strategies; but also indicating the importance of bioactivity introduction in protoplast culture platforms. As a proof of concept, single protoplasts encapsulated in supramolecular microgels could be engineered as well. This might offer potential advantages for the future by simplifying single‐cell level assays without the necessity for manual separation in future protoplast gene editing applications.

## 4 Experimental Section

### Materials

All compounds were used as received, unless stated otherwise. Murashige and Skoog basal medium, Murashige and Skoog (MS) medium including vitamins, Kao & Michayuk medium, Kao Vitamins, cellulase R‐10, macerozyme R‐10, pectolyase, timentin, carbenicillin, mannitol, sorbitol, glucose, sucrose, fructose, ribose, and xylose, were purchased from Duchefa Biochemie. Agar, mannose, rhamnose, cellobiose, sucrose, coconut water, myo‐inositol, thiamine, KH_2_PO_4_, NaOH, KCl, CaCl, Na‐Pyruvate, citric acid, fumaric acid, malic acid, ammonium hydroxide, 1‐Naphthaleneacetic acid (NAA), zeatin, 2,4‐dichlorophenoxyaetic acid (2,4‐D), 2‐(N‐morpholino)ethanesulfonic acid (MES), calcofluor white (CFW), fluorescein diacetate (FDA), 1H,1H,2H,2H‐Perfluoro‐1‐octanol (PFO) were purchased from Sigma–Aldrich. Silicone elastomer base 184 (polydimethylsiloxane; PDMS base) and silicone elastomer curing agent were purchased from Sylgard. 1H,1H,2H,2H‐perfluorooctyl‐triethoxysilane and HFE‐7500 3M Novec engineered fluid (Fluorinated oil) were purchased from Fluorchem UK. Pico‐Surf surfactant was purchased from Sphere Fluidics. Mineral oil was purchased from Merck. SU‐8 3000 photoresist was purchased from Microresist Technologies. Ibidi 15 µ‐Slide Angiogenesis glass bottom culture plates were purchased from Ibidi, and 125 mL Nalgene Erlenmeyer culture flasks from ThermoFisher Scientific.

### Reagent Setup

MS medium: basal culture medium was prepared based on an adapted protocol provided by RIKEN Experimental Plant Division for modified Linsmaier and Skoog medium. 4.4 g L^−1^ Murashige and Skoog basal medium, 30 g L^−1^ sucrose, 0.1 g L^−1^ myo‐inositol, 0.2 g L^−1^ KH_2_PO_4_, and 0.2 mg L^−1^ 2,4‐D. pH was adapted to 5.8 with 1 m NaOH and autoclaved. Filtered 1 mg L^−1^ thiamine, 0.1 mg mL^−1^ timentin, and 0.1 mg mL^−1^ carbenicillin were added.

Solid MS medium: MS medium including 0.8 w/v% agar, autoclaved. Filtered 0.1 mg mL^−1^ timentin, and 0.1 mg mL^−1^ carbenicillin were added.

Enzyme digestion solution: medium was prepared based on an adapted protocol reported by Lei et al. and Negrutiu and coworkers.^[^ ^54^, ^55^ ^]^ Freshly prepared 1 w/v % cellulase R‐10, 0.5 w/v% macerozyme R‐10, 0.1 w/v% pectolyase, 20 mm MES, 0.5 m mannitol, 20 mm KCl, and 10 mm CaCl_2_. pH was adapted to 5.8 with 1 m NaOH and filter sterilized.

Regeneration medium K8P‐L/8p2c: regeneration medium was prepared based on an adapted protocol of Kao and Michayluk for 8p medium, and Kirchhoff and co‐workers for 8p2c medium.^[^ ^58^, ^59^ ^]^ 1% Kao Vitamins, 2 v/v% coconut water, 0.35 mm glucose, 0.07 mm sucrose, 0.14 mm fructose, 0.17 mm ribose, 0.17 mm xylose, 0.14 mm mannose, 0.14 mm rhamnose, 0.07 mm cellobiose, 0.10 mm sorbitol, 0.14 mm mannitol, 0.5 mg L^−1^ zeatin, 1.0 mg L^−1^ NAA, 0.2 mg L^−1^ 2,4‐D, 10% K10 stock + Iron (for 100 mL: Kao & Michayluk medium, 0.02 g Na‐Pyruvate, 0.04 g citric acid, 0.04 g malic acid, 0.04 fumaric acid, 2 mL NH_4_OH ammonium hydroxide, 0.1 mm myco‐inositol, and fill up with Milli‐Q water). pH was adapted to 5.8 with 1 m NaOH and filter sterilized.

### Maintenance of Cell Line

*Nicotiana tabacum* L. cv. Bright Yellow 2 (tobacco BY‐2) cells genetically modified with the fluorescent protein DsRed (BY‐2 glyco DsRed ERH #5; transgenic tobacco BY‐2 cells producing glycosylated DsRed) were kindly obtained via Prof. Dr. Stefan Schillberg (Fraunhofer Institute for Molecular Biology and Applied Ecology IME). They were grown in suspension culture under sterile conditions in liquid MS medium in 125 mL Erlenmeyer flasks with constant orbital agitation at 110 rpm (Minitron, Infors HT) in the dark at 25 °C. Subcultivation was performed at 7‐day intervals by transferring 10 mL of suspension culture into 40 mL fresh medium. This equals 10–20% packed cell volume (PCV). Stock cultures were maintained as callus tissue for four weeks on solid MS medium and were subcultured monthly. Cell suspensions were established by resuspending 2 cm callus pieces in liquid MS medium.

### Isolation of Single Tobacco Protoplasts

Protoplasts were enzymatically isolated from 10–20% PCV actively growing suspension culture, 3 days after subculture. For this, 10 mL of the cell suspension was spun down (300 × g, 5 min). The freshly prepared enzyme digestion solution was heated at 55 °C for 10 min, cooled down, pH adapted to 5.8 with 1 m NaOH, and filter sterilized prior to use. The supernatant of the spun‐down cells was removed, and the cells were resuspended in 15 mL enzyme digestion solution, whereafter the cells were incubated for 3 h in the dark at 25 °C. The enzyme solution containing protoplast was gently filtered using a 100 µm mesh and centrifuged (80 × g, 4 min) to form a pallet. The supernatant was carefully removed, and the protoplasts were washed with regeneration medium and filtered once more, whereafter the cells were counted using an automated cell counter. The protoplast suspension was centrifuged once more (80 × g, 4 min) and resuspended in regeneration medium at the desired concentration.

### Protoplast Staining

Viability: Protoplast viability was assessed with a FDA staining (1 g mL^−1^ stock in acetone; 0.2 v/v% in milli‐Q work solution) one day after isolation. Untreated Tobacco BY‐2 cells in suspension were used as a control. The FDA fluorescence was determined using a Leica SP8 X fluorescence confocal microscope (Leica Microsystems) using HC PL APO CS2 objectives (20x/0.75, 40x/0.95). Images were processed and manually analyzed using ImageJ.

Cell wall staining: The success of the cell wall removal was assessed by staining cellulose with 0.2 v/v% Calcofluor White (CFW) in Milli‐Q for 10 min directly after protoplast isolation. Untreated Tobacco BY‐2 cells in suspension were used as a control. The CFW fluorescence was determined using a Leica SP8 X fluorescence confocal microscope (Leica Microsystems) using HC PL APO CS2 objectives (20x/0.75, 40x/0.95). Images were processed using ImageJ.

### 2D and 3D UPy‐Hydrogel Formation

Hydrogel pre‐solutions of B‐type and M‐type UPy‐molecules were prepared separately. M‐type component (UPy‐Glycine +/‐ UPy‐cRGD) was dissolved at twice the desired end concentration for 20 min at 70 °C in an alkaline solution containing 80 or 160 mm NaOH for the preparation of hydrogels with < 5 w/v% or ≥ 5 w/v%, respectively. The pH was brought to 5.8 by the addition of 1 m HCl or 2 m HCl, respectively, to the base. The solution was diluted one time with regeneration medium and UV‐sterilized for 20 min. The B‐type molecule was dissolved at three times the desired end concentration for 1 h at 70 °C in an alkaline solution containing 80 mm NaOH. The pH was brought to 5.8 by the addition of 1 m HCl. The solution was UV‐sterilized for 20 min. For 2D gels, the solution was diluted to the final desired concentration with regeneration medium. For 3D gels, the solution was diluted to the final desired concentration with BY‐2 protoplasts in regeneration medium to obtain an end concentration of 2 million cells per mL. Both B‐type and M‐type solutions with or without cells were mixed at a 1:1 volume ratio by pipetting, and 15 µL of the UPy‐mixture was transferred to an Ibidi‐slide. For 3D, the gels were allowed to gelate at 25 °C for 2 h, whereafter 30 µL of regeneration medium was added to the gels. For 2D hydrogels, the gels were allowed to gelate at 25 °C for at least 2 h whereafter 30 µL of regeneration medium with 1×10^6^ protoplasts per mL was added to the gels. This equals the amount of cells embedded in the 3D hydrogel. As control, liquid suspension cultures without matrix were used with equal amount of protoplasts. Protoplasts were cultured at 25 °C in the dark for 11 days, and the medium was replaced once a week. Growth was assessed by visualizing protoplast morphological changes taking brightfield images at day 4 and 11. Manually analysis were performed in ImageJ by encircling the protoplasts and measuring the object (protoplast) area and eccentricity (circularity). Protoplast viability was assessed at day 1 with a FDA staining (EVOS XL Core Imaging System and Leica SP8 X fluorescence confocal microscope; Leica Microsystems).

### Rheology ‐ 2.0 w/v% UPy‐Hydrogels for 2D and 3D Culture

Hydrogels of 2.0 w/v% were measured during gelation on the rheometer. The measurements were carried out on a TA Instruments Dynamic Hybrid Rheometer 3 in a 20 mm aluminum cone‐plate (2.007°) geometry with a truncation gap of 56 µm. A solvent trap was used to minimize sample drying. Samples were loaded at 10 °C and allowed to equilibrate for a short period. To measure the linear regime (G0), the sample was heated to 25 °C, and the complex modulus G^*^ was measured for 19 h by applying an oscillating deformation of amplitude γ = 0.01 at frequency ω = 1 rad s^−1^. Subsequent frequency sweep measurements were performed at ω = 0.01 to 100 rad s^−1^, at a strain of γ = 0.01. Stress‐relaxation was measured by applying a strain of γ = 0.15 with a strain rise time of 0.05 s and monitoring the stress for 10 000 s. The data were normalized using the highest stress generated during the experiments (always after 0.05s, disregarding data that was shorter than the strain rise time). Strain‐sweep measurements were performed at strains between γ = 0.0001 and γ = 10 with a frequency of ω = 1 rad s^−1^. Results are shown in Figure S2C (Supporting Information).

### Rheology ‐ 1.0 w/v% UPy‐Hydrogel for 2.5D Culture

Hydrogels of 1.0 w/v% were measured after gelation overnight at 4 °C. Rheological measurements were carried out on a TA Instruments Dynamic Hybrid Rheometer 3 equipped with an 8 mm flat steel plate‐plate geometry with a gap of 850 µm. Low viscosity silicon oil (47 V 100, RHODORSIL) was used around the hydrogel to minimize sample drying. Samples were loaded at 25 °C, after which the complex modulus G^*^ (γ = 0.01, ω = 1 rad s^−1^) was measured for 5 min to ensure that samples were at a stable plateau modulus and not altered or damaged during loading. Subsequent frequency sweep measurements were performed at ω = 0.01 to 100 rad s^−1^, at a strain of γ = 0.01. Stress‐relaxation was measured by applying a strain of γ = 0.15 with a strain rise time of 0.05 s and monitoring the stress for 10 000 s. The data were normalized using the stress generated after 0.6 s (disregarding data that was shorter than the strain rise time). Strain‐sweep measurements were performed at strains between γ = 0.0001 and γ = 10 with a frequency of ω = 1 rad s^−1^. Results are shown in Figure S4C (Supporting Information).

### Protoplast Culture in 2.5D

Protoplasts were isolated, and 1.0 w/v% hydrogels were prepared as described earlier. Supramolecular UPy‐fibers enriched with the UPy‐cRGD additive were prepared by dissolving the M‐type UPy‐building blocks along with UPy‐cRGD at twice the desired end concentration (Table S2, Supporting Information) in 80 mM NaOH at 70 °C for 20 min. The pH was brought to 5.8 by the addition of 1 m HCl. The solution was diluted one time with regeneration medium and UV‐sterilized for 20 min. Thereafter, the stock fiber solution was further diluted to the right concentration. For each concentration fibers, 1 × 10^6^ protoplasts per mL were transferred to an Eppendorf and spun down (80 x g, 4 min). The protoplasts were resuspended in the UPy‐fiber solution, whereafter 30 µL of this mixture was added on the hydrogel or an Ibidi‐well. Protoplasts were cultured at 25 °C in the dark to allow regeneration for 4 days. Growth was assessed by visualizing protoplast morphological changes, taking brightfield images at day 1 and 4 (EVOS XL Core Imaging System and Leica SP8 X fluorescence confocal microscope; Leica Microsystems). Manual analysis was performed in ImageJ by encircling the protoplasts and measuring the object (protoplast) area and eccentricity (circularity).

### Microfluidic Device Production

Microfluidic devices were produced from polydimethylsiloxane (PDMS) molds using soft lithography. Photomasks for soft photolithography were ordered from CAD/Art Services, Inc. (Bandon, Oregon). PDMS molds were produced by spin‐coating wafers with SU‐8 3000 photoresist (Microresist Technology) according to the manufacturer's protocol to obtain 70 µm of channel height. Microfluidic devices were fabricated by mixing polydimethylsiloxane (PDMS) base and curing agent at a ratio of 10:1 w/w%. After the air bubble was removed with vacuum, the mixture was poured onto a master silicon wafer containing the device layout (Figure S5, Supporting Information), and the mixture was cured at 65 °C for 3 h. After curing, the PDMS was carefully removed from the wafer, and 1 mm holes were punched for the inlets and outlets. The PDMS was bonded channels‐down to glass slides to yield closed microchannels via OH‐terminated by exposure to plasma (Emitech K1050X), whereafter the devices were incubated at 65 °C for 1 h to increase binding. Finally, channels were treated with 5 v/v% silane in fluorinated HFE‐7500 oil, incubated for one hour at 65 °C, flushed with HFE‐7500 oil, and incubated overnight at 65 °C for thermal bonding.

### Microgel Formation

Protoplasts encapsulated in microgels based on supramolecular building blocks were generated using a tip‐loading approach as previously described in Sinha et al., 2019, and Rovers et al., 2024.^[^ ^53^, ^60^ ^]^ Supramolecular hydrogel pre‐solutions of B‐type and M‐type UPy‐molecules with or without protoplasts were prepared separately as described previously. The solutions were disinfected by exposing them to UV‐light for 20 min, as well as the tubes, Eppendorfs, and microfluidic chip. For protoplasts encapsulated in microgels, the protoplasts were suspended in regeneration medium, and this cell suspension was added to the B‐type solution with a cell concentration of 2000 cells per µL. Next, the B‐type and M‐type pre‐solutions were separately drawn into a 200 µL pipette tip and loaded according to Figure S5 (Supporting Information) to the inlets in the PDMS chip as had been described.^[^ ^60^ ^]^ Syringes and tubing filled with mineral oil were used as a hydraulic system to dispense the liquids from the syringes, driven by computer‐controlled pumps (neMESYS microfluidic pump, Cetoni). HFE‐7500 with 2.5 v/v% Pico‐Surf was flushed into the oil inlet to form microgels. Microgel production was started with oil flow at a speed of 10 µL min^−1^ to prefill the channels of the microfluidic devices. The flow of the hydrogel pre‐solutions was started simultaneously at 5 µL min^−1^. The flow of the oil was increased to 30 µL min^−1^ once the hydrogel pre‐solutions started to mix at the droplet formation point. The microgels were collected in an Eppendorf tube from the outlet, and regeneration medium was poured on top of the emulsion to prevent evaporation of the HFE oil. Protoplasts encapsulated in microgels were incubated for 3 h in the dark at 25 °C to allow gelation of the microgel. Thereafter, the microgels were isolated by demulsification with 20 v/v% PFO in HFE‐7500 oil. Microgels were collected and resuspended in fresh regeneration medium.

### Microgel Stability

Microgels (2.0 w/v%) were fabricated using the previously described method. Then, microgels were isolated from the oil phase and resuspended in regeneration medium with pH values of 2, 5.8, and 10. The microgel diameter was determined from brightfield images at day 1, 2, 8, and 11 for all conditions. To assess microgel stability, statistical analysis was performed, wherein the data was first checked for normal distribution using the Kolmogorov–Smirnov test. Subsequently, a nonparametric Kolmogorov–Smirnov test was applied to compare cumulative distributions. GraphPad Prism 8.0.2 software was utilized for conducting the statistical analysis.

### Statistical Analysis

Statistical analyses were carried out using GraphPad Prism 8.0.2 software. For all experiments, normality was assessed using the Shapiro–Wilk test. Data related to the protoplast area and circularity were obtained from brightfield images using ImageJ. Since these conditions did not follow a normal distribution, the Kolmogorov–Smirnov test was applied to identify statistically significant differences between the conditions. For the 2.5D experiments on hydrogels, a Kruskal–Wallis test was applied. Data related to microgel stability were analyzed using a nonparametric Kolmogorov–Smirnov test. In all cases, differences were considered as statistically significant when *P* < 0.05.

## Conflict of Interest

The authors declare no conflict of interest.

## Supporting information



Supporting Information

## Data Availability

The data that support the findings of this study are available from the corresponding author upon reasonable request.
