# Supplementary material for: Using a Supramolecular Approach to Engineer Modular Hydrogel Platforms for Culturing Protoplasts – from General Tissue Engineering to Cellular Agriculture
Source: Adv Biol (Weinh). 2025 Jun 4;9(11):e00690. doi: 10.1002/adbi.202400690 (PMC12624825; doi:10.1002/adbi.202400690)
Supplement: Supplementary file 1 — Supporting Information [file ADBI-9-e00690-s001.docx]

Supporting Information

Using a supramolecular approach to engineer modular hydrogel platforms for culturing protoplasts – from general tissue engineering to cellular agriculture

Maritza M. Rovers,^1,2^ Erik J. Slootweg,^3^ Ferdinand C.O. Los,^3^ Patricia Y.W. Dankers ^1,2,4^*

**Table S1:** Overview of the different studied hydrogel compositions for 2D, 2.5D and 3D protoplast culture.

| *Sample ID* | *B-type* | | | *M-type: UPy-Glycine + UPy-cRGD* | | | | | | *Total in hydrogel* |
| --- | --- | --- | --- | --- | --- | --- | --- | --- | --- | --- |
|  | mM \| w/v% \| mol% | | | mM \| w/v% \| mol% | | | | | |  |
|  |  | | |  | | | | | |  |
| 1. w/v% \|   pristine | 0.09 | 0.10 | 1.18 | 7.58 | 0.91 | | | 98.82 | | 7.67 mM \| 1.01 w/v% |
| 2.0 w/v% \| pristine | 0.18 | 0.20 | 1.18 | 15.19 | 1.82 | | | 98.82 | | 15.37 mM \| 2.02 w/v% |
| 2.0 w/v% \|  + UPy-cRGD | 0.18 | 0.20 | 1.18 | UPy-Glycine | | 14.69 | 1.76 | | 95.57 | 15.37 mM \| 2.05 w/v% |
|  |  |  |  | UPy-cRGD | | 0.5 | 0.09 | | 3.25 |  |


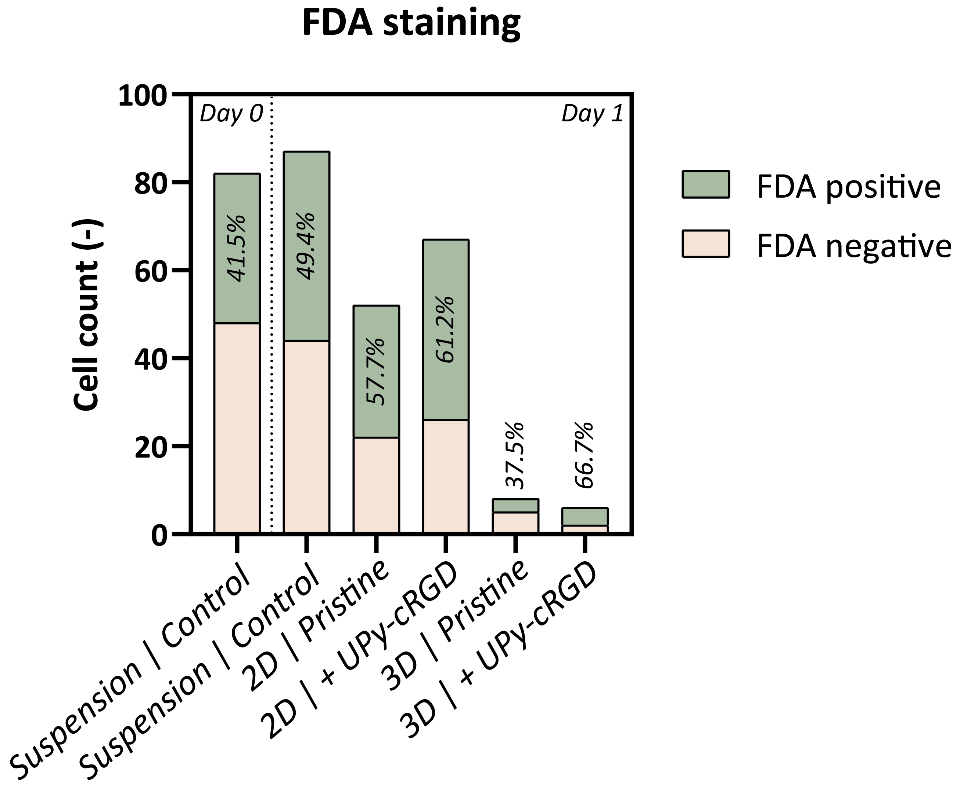


**Figure S1: Quantified FDA staining.** Quantification of FDA-stained protoplasts as a percentage (%) of the total protoplast population, with FDA-positive cells shown in green and FDA-negative cells in soft orange. Protoplasts were imaged in suspension within a well directly after isolation at day 0 and after 1 day of culture in suspension, or on 2D and 3D hydrogels, without (pristine) or with UPy-cRGD.


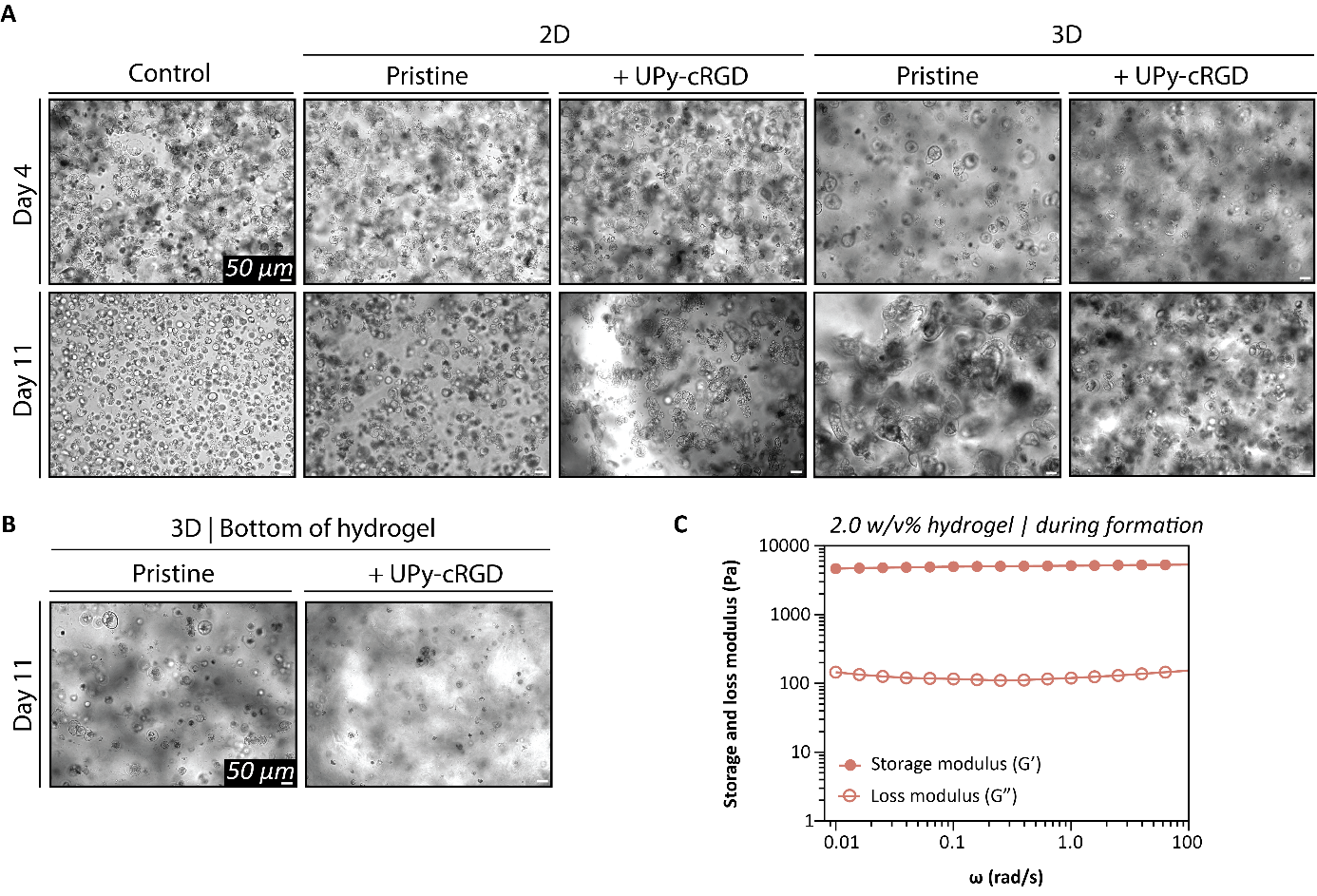


**Figure S2: Total overview of protoplast cultured in 2D and 3D.** **A)** Protoplast regeneration potential was assessed by morphological changes from spherical to a chain-like shape, for both 2D and 3D culture methods with and without UPy-cRGD functionalization, at day 4 and 11. **B)** Heterogeneity in cellular morphology was observed for protoplasts cultured in 3D hydrogels at day 11. Protoplasts at the bottom of the hydrogel exhibited smaller and irregular shapes, suggestive of cellular demise. **C)** Rheological measurement during 2.0 w/v% hydrogel formation showing storage (G’) and loss (G”) moduli in an angular frequency sweep plot. All scale bars represent 50 μm.


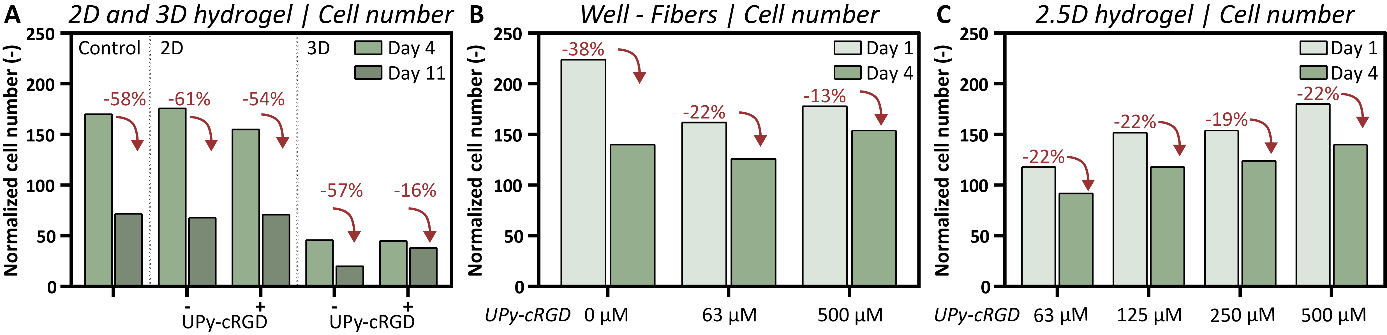


**Figure S3: Normalized cell number of protoplasts cultured in 2D, 3D, and 2.5D. A)** Normalized cell number of protoplasts cultured for 4 and 11 days under different conditions: suspension culture without a supramolecular matrix (control), or 2D culture on hydrogels and 3D encapsulation in hydrogels, both with or without UPy-cRGD. **B)** Normalized cell number of protoplasts cultured in a well with UPy-cRGD enriched supramolecular fibers in solution for 1 and 4 days. **C)** Normalized cell number of protoplasts cultured on a hydrogel with UPy-cRGD enriched supramolecular fibers in solution for 1 and 4 days.

**Table S2:** Overview of the different formulated supramolecular RGD-enriched fibers for 2.5D protoplast culture.

| *Sample ID:*  *μM UPy-cRGD in fiber* | *UPy-Glycine* | | | *UPy-cRGD* | | | *Total in fiber* | |
| --- | --- | --- | --- | --- | --- | --- | --- | --- |
|  | μM \| w/v% \| mol% | | | μM \| w/v% \| mol% | | | μM \| w/v% | |
|  |  | | |  | | |  |  |
| 0 μM | 0 | 0 | 0 | 0 | 0 | 0 | 0 | 0 |
| 63 μM | 127 | 0.015 | 66.97 | 63 | 0.01 | 33.03 | 190 | 0.03 |
| 125 μM | 253 | 0.03 | 66.97 | 125 | 0.02 | 33.03 | 378 | 0.05 |
| 250 μM | 507 | 0.06 | 66.97 | 250 | 0.04 | 33.03 | 757 | 0.10 |
| 500 μM | 1014 | 0.12 | 66.97 | 500 | 0.09 | 33.03 | 1514 | 0.21 |


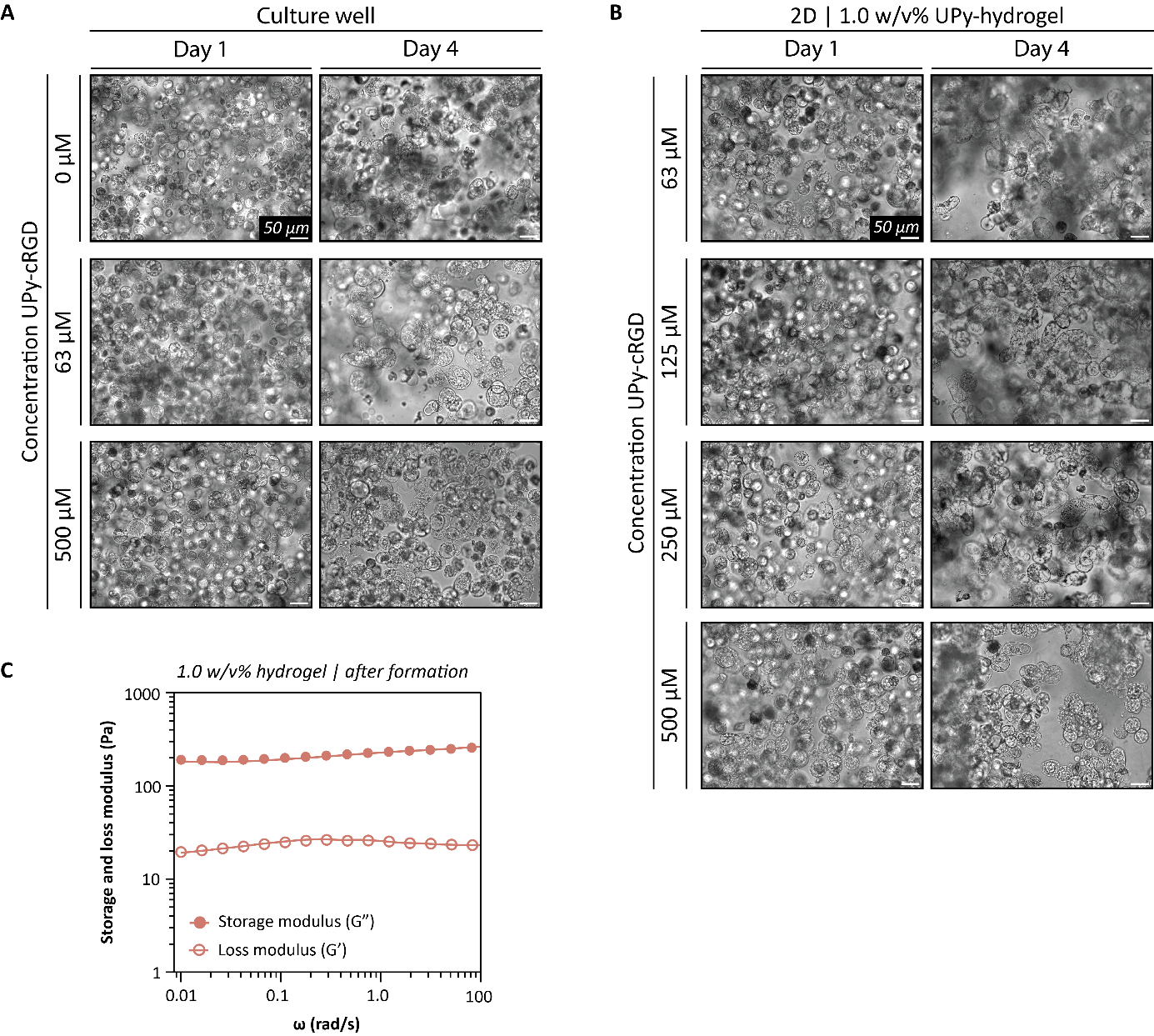


**Figure S4:** **Overview of 2.5D protoplast culture with UPy-cRGD enriched supramolecular fibers in solution.** **A)** Brightfield images of protoplasts cultured in a well with supramolecular fibers containing 0 μM, 63 μM, or 500 μM UPy-cRGD at 1 and 4 days. **B)** Brightfield images of protoplasts cultured on a 2D 1.0 w/v% hydrogel with supramolecular fibers containing 63 μM, 125 μM, 250 μM or 500 μM UPy-cRGD at 1 and 4 days. **C)** Rheological measurement after 1.0 w/v% hydrogel formation showing storage (G’) and loss (G”) moduli in an angular frequency sweep plot. All scale bars represent 50 μm.

**Table S3:** Overview of the different formulated supramolecular microgels used in this study.

| *Sample ID* | *B-type* | | | *M-type: UPy-Glycine* | | | *Total in microgel* |
| --- | --- | --- | --- | --- | --- | --- | --- |
|  | mM \| w/v% \| mol% | | | mM \| w/v% \| mol% | | |  |
|  |  | | |  | | |  |
| 1.25 w/v% | 0.11 | 0.13 | 1.18 | 9.48 | 1.13 | 98.82 | 9.59 mM \| 1.26 w/v% |
| 2.0 w/v% | 0.18 | 0.20 | 1.18 | 15.19 | 1.82 | 98.82 | 15.37 mM \| 2.02 w/v% |


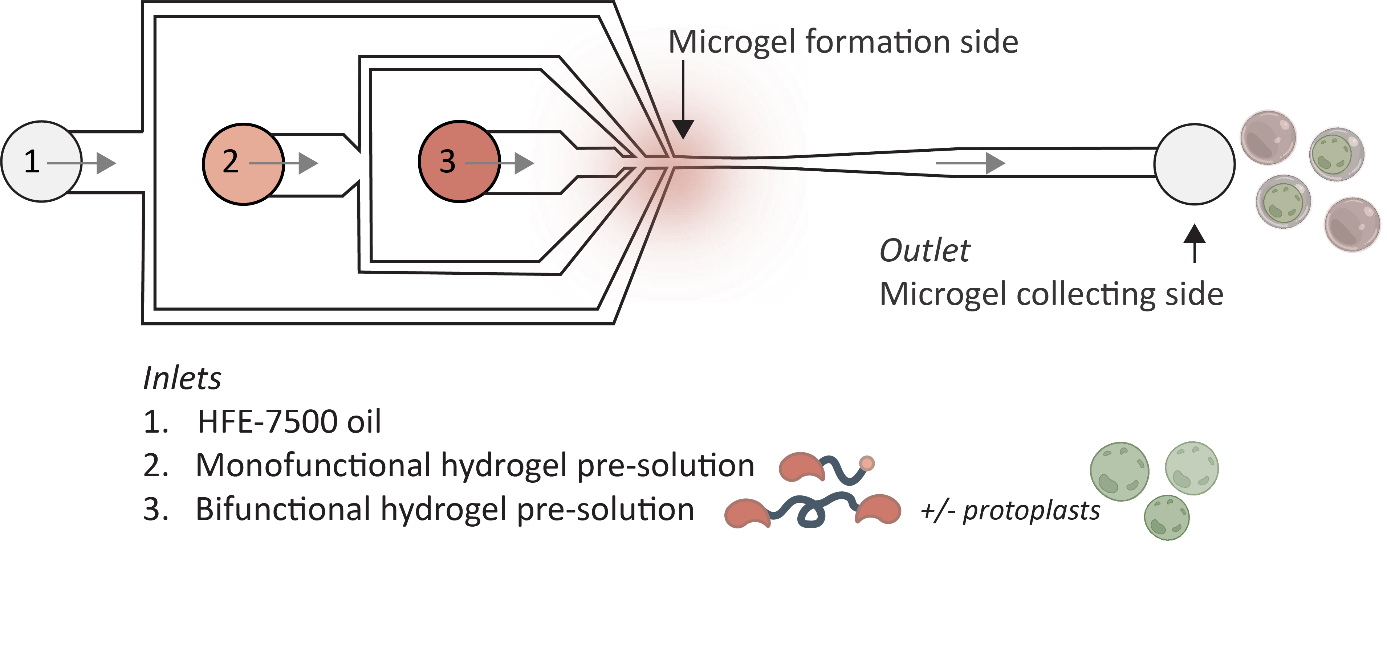
**Figure S5:** Schematic representation of the microfluidic chip lay-out to formulate (protoplast encapsulated in) microgels.
